# PB.41: Audit of screening-detected breast cancers with discordant interpretations on double-read screening mammography

**DOI:** 10.1186/bcr3541

**Published:** 2013-11-08

**Authors:** B Batohi, R Rahim, M Adejolu, M Michell

**Affiliations:** 1 King's College Hospital, London, UK

## Introduction

Discordant breast cancers are identified following differing interpretations on double-read screening mammograms. The aim of this audit was to analyse the mammographic features of discordant breast cancers to improve reader sensitivity.

## Methods

The screening programme database was used to identify all breast cancers that were diagnosed from 1 April 2010 to 31 March 2012. Discordant cases were reviewed at a consensus meeting in which the mammographic sign, size, site, parenchymal pattern and final histology were recorded.

## Results

Fifty-seven of a total of 670 cancers had discordant reads. The most common mammographic abnormality was spiculated masses (30%). Microcalcifications accounted for 25% of cases. Eighty-five per cent of the abnormalities were small (i.e. ≤15 mm). No single view was noted to be more likely to reveal the abnormality. The histology results showed no difference in distribution of tumour type compared with nondiscordant tumours. The second reader identified 69% of the discordant cancers.

## Conclusion

A total 8.5% of the screen-detected cancers in this audit were discordant-read cancers, which corresponds with published findings [1]. The mammographic features of discordant cancers were similar to nondiscordant cancers. The study supports the continued use of two readers to maximise sensitivity [2].
